# Expression Proteomics Predicts Loss of RXR-γ during Progression of Epithelial Ovarian Cancer

**DOI:** 10.1371/journal.pone.0070398

**Published:** 2013-08-06

**Authors:** Rajkumar S. Kalra, Sharmila A. Bapat

**Affiliations:** National Centre for Cell Science, NCCS Complex, Pune University Campus, Ganeshkhind, Pune, Maharashtra, India; University of Alabama at Birmingham, United States of America

## Abstract

The process of cellular transformation involves cascades of molecular changes that are modulated through altered epigenetic, transcription, post-translational and protein regulatory networks. Thus, identification of transformation-associated protein alterations can provide an insight into major regulatory pathways activated during disease progression. In the present protein expression profiling approach, we identified differential sets of proteins in a two-dimensional gel electrophoresis screen of a serous ovarian adenocarcinoma progression model. Function-based categorization of the proteins exclusively associated with pre-transformed cells identified four cellular processes of which RXR-γ is known to modulate cellular differentiation and apoptosis. We thus probed the functional relevance of RXR-γ expression and signaling in these two pathways during tumor progression. RXR-γ expression was observed to modulate cellular differentiation and apoptosis in steady-state pre-transformed cells. Interestingly, retinoid treatment was found to enhance RXR-γ expression in transformed cells and sensitize them towards apoptosis *in vitro*, and also reduce growth of xenografts derived from transformed cells. Our findings emphasize that loss of RXR-γ levels appears to provide mechanistic benefits to transformed cells towards the acquisition of resistance to apoptosis hallmark of cancer, while effective retinoid treatment may present a viable approach towards sensitization of tumor cells to apoptosis through induction of RXR-γ expression.

## Introduction

A contemporary view of tumorigenesis is that transformation results as a multi-step process involving genetic, epigenetic, cellular and tissue-associated changes [1], [2], [3]. These effect alterations in several regulatory and functional networks within the cell that lead to a progressive acquisition of capabilities of self-sufficiency in growth signals, insensitivity to anti-growth signals, unlimited replicative potential, evasion of apoptotic signals, tissue invasion and metastasis, and sustained angiogenesis [4]. More lately, energy metabolism reprogramming and evading immune destruction have received recognition as additional hallmarks of transformation [5].

Epithelial Ovarian Cancer (EOC) is recognized as the fifth most common cancer and the highest cause of cancer-related deaths among woman [6], [7]. A limitation in EOC studies is the lack of identification of pre-neoplastic lesions that lead to rapid and aggressive metastasis, at which stage the disease is most frequently diagnosed. This is further made more complex from recent findings that suggest high-grade EOC to originate in the fallopian tube epithelia, in contrast to the classical opinion of the ovarian surface epithelia being the cell of origin [8], [9]. Though contemporary proteome analyses provide a dynamic and efficient source of identification of tumor suppressors, oncogenes, cancer diagnostics and therapeutics [10], [11], [12], [13], an extended understanding of the multi-step transformation events in EOC vis-a-vis altered molecular expressions among transformed and pre-transformed cells remains to be resolved.

The present study is based on proteomic profiling of an *in vitro* model of serous ovarian adenocarcinoma (SeOvCa) established earlier in our lab [14]. Briefly, we had established several single-cell clone derived cultures from the malignant ascites of a Grade IV serous ovarian adenocarcinoma patient. Nineteen of these underwent spontaneous immortalization and were established as continuous lines. The A4 clone was one of these clones. In its initial passages, it was seen to be slow-cycling and non-tumorigenic; however, around passage 20–25 it transformed into an aggressively tumorigenic clone with metastatic capabilities. This data suggests that early A4 cells, although lacking tumorigenecity had already acquired some of the features of transformation. Hence we referred to these as being pre-transformed (A4-P), while the transformed cells derived from A4-P cells were termed as A4-T. This provided us a suitable progression model of two functionally discrete cell groups derived from a single clone in the tumor. Proteome profiles of this A4 progression model resolved through 2-Dimension Gel Electrophoresis (2DE) followed by MS (MALDI-TOF/TOF) led to the derivation of specific protein groups based on their exclusive and differential expression patterns.

Characterization of the functional networks defined by such proteins provided a clear insight into altered cellular functionality and major pathways involved in ovarian cell transformation. Of these, RXR-γ modulated cellular differentiation and apoptosis were exclusive to the pre-transformed cells. Modulation of retinol metabolism has been suggested in association with EOC progression [15], [16] in which decreased levels of CRBP1 (cellular retinol-binding protein-1) are considered a crucial step in progression of the transformation process [17]. However, the precise relevance of RXR-γ signaling remains largely uncharacterized. We resolved its functional role in the transformed cells of our progression model through induction of expression by treatment with selective retinoids including 9*Cis*-Retinoic acid (CRA), Adapalane (ADA) and 4-[(E)-2-(5,6,7,8-Tetrahydro-5,5,8,8-tetramethyl-2-naphthalenyl)-1-propenyl] benzoic acid (TTNPB). Such modulation of cellular differentiation and apoptosis by RXR-γ in SeOvCa further extends our current understanding of cellular transformation.

## Results

### Comparative protein expression analysis of A4-P and A4-T epithelial ovarian cancer progression model

**Figure 1:**
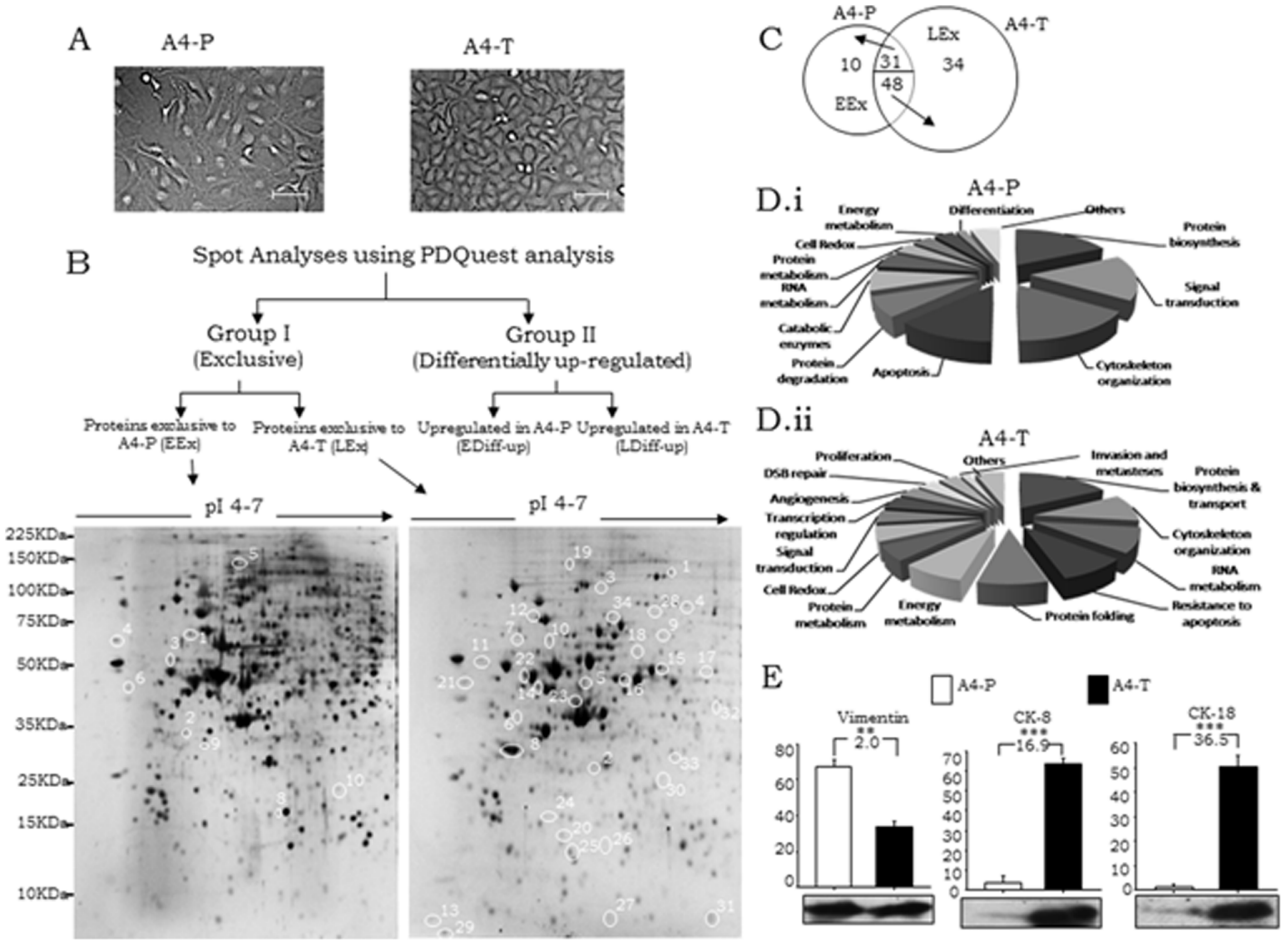
Expression profiling of the proteome of serous ovarian adenocarcinoma progression model. A. Morphological differences between A4 pre-transformed (A4-P) and A4 transformed (A4-T) cells (Bar 100μ). B, Analytical pipeline; Lower Panel-Representative gel images showing exclusively expressed proteins in A4-P cells (left – EEx) and A4-T cells (right – LEx). C, Venn diagram showing proteins categorized under all 4 sub-groups. D.i-ii, Pie diagram showing molecular functionality and pathways associated with identified A4-P and A4-T proteins respectively. E, Quantitative protein expression of Vimentin, Cytokeratin-8 and Cytokeratin-18; data shown are representative of 3 separate experiments depicted as mean ± SEM *p<0.05, **p<0.01, ***p<0.00.

The functionally different A4-P and A4-T epithelial ovarian cancer cells exhibit a distinct phenotype, with the former being spindle-shaped while the latter appear epithelial-like in morphology (Fig. 1A). 2-DE gels were prepared using proteome samples of A4-P and A4-T cells. Two technical sets of 2-DE analytical gels were prepared from each phenotype in each experiment, which was carried out in triplicate (total 6 replicates) and silver-stained. Scanned images were processed using PDQuest and proteins with differential expression were annotated. An average, 400–500 differential protein spots were thus demarcated. Annotation of spots led to the derivation of protein sets based on their expression patterns in each cell type. Towards identification of differential protein expression, selected protein spots were digested and mass spectra was generated in MS/MS analyses. GPS Explorer software (v.3.6) was used to submit the combined MS and MS/MS data from MALDI-TOF/TOF to Mascot against SwissProt database. For all proteins thus analysed, reasonable sequence coverage, low index of mass errors and high confidence interval (CI ≥95%) were obtained.

### Derivation of the protein groups based on expression pattern

MS/MS based protein identification led to derivation of two groups of differentially expressed proteins (Fig. 1B). *Group I* comprised of proteins that were expressed qualitatively (exclusively) in either A4-P or A4-T (termed as EEx and LEx proteins respectively), while *Group II* includes proteins expressed at quantitatively different levels (minimum two-fold differential expression between the two cell types). Both groups were further divided into two sub-groups based on their expressions in respective cell types. Annotation of qualitative and quantitative expressions was performed within each replicative set of A4-P and A4-T cells. A total of 10 and 34 *Group I* proteins and, 31 and 48 *Group II* proteins were identified as being expressed in A4-P and A4-T cells respectively (Fig. 1C; Table S1). Tables 1 & 2 lists the identified *Group I* and *Group II* proteins with specific spot numbers, molecular and functional description along with the details of match peptides, protein score, sequence coverage (%) and relative expression fold-change.

**Table 1:** Details of proteins identified through 2DE followed by MALDI-TOF (MS/MS) analysis in Group I.

Sub-group I. Proteins qualitatively expressed in A4-P cells.												
Spot No.	Accession No.	Description of identified proteins	Function	SwissProt Accession	Gene name	Gene ID	Mass (Da)/PI	Peptide matched	Score	(%) Sequence coverage	RMS (ppm)	Validation method
**1**	P07339	Cathapsin D precursor	Cell death/proteolysis	CATD_HUMAN	CTSD	1509	44524/6.10	11	244	37	43	2D
**2**	P29992	Guanine nucleotide binding protein G(y) alpha subunit	Protein amino adic ADP-ribosylation	GNA11_HUMAN	GNA11	2767	42097/5.11	7	49	20	49	2D
**3**	Q9P2J3	Kelch like protein -9	Ubl conjugation pathway	KLHL9_HUMAN	KLHL9	55958	69383/5.92	13	39	23	73	2D
**4**	Q8NEV4	Myosin 3A	Autophosphorylation and response to stimulus	MYO3A_HUMAN	MYO3A	53904	185966/9.0	21	41	14	64	2D
**5**	Q8WXW3	Progestrone induced blocking factor-1	Progestrone mediator	PIB1_HUMAN	PIBF1	10464	89719/5.77	13	43	20	30	2D
**6**	P48443	RXR gamma retinoic acid receptor	Transcriptional regulation, differentiation and proliferation	LMO7_HUMAN	RXRG	6258	50838/7.55	11	39	19	69	2D: IB
**7**	Q15293	Reticulocalbin 1 precursor	endoplasmic reticulum lumen	RCN1_HUMAN	RCN1	5954	38866/4.86	8	68	23	22	2D
**8**	P08758	Annexin A5	anti-apoptosis/blood coagulation	ANXA5_HUMAN	ANXA5	308	35783/4.94	18	243	66	23	2D
**9**	Q13162	Peroxiredoxin 4	I-kappaB phosphorylation/cell redox homeostasis	PRDX4_HUMAN	PRDX4	10549	30521/5.86	11	208	49	11	2D
**10**	P09493	Tropomyosin 1 α chain	Actin binding and cellular dynamics	TMP1_HUMAN	TPM1	7168	32689/4.69	11	146	21	10	2D

Sub-group II. Proteins qualitatively expressed in A4-T cells.												
Spot No.	Accession No.	Description of identified proteins	Function	SwissProt Accession	Gene name	Gene ID	Mass (Da)/PI	Peptide matched	Score	(%) Sequence coverage	RMS (ppm)	Validation method
**1**	P13010	ATP-dependent DNA helicase II,	DNA Repair and telomerase maintenance	KU86_HUMAN	XRCC5	7520	82521/5.55	17	194	24	20	2D: IB
**2**	Q9Y696	Chloride intracellular channel protein 4	Cell diffrentiation and chloride transport	CLIC4_HUMAN	CLIC4	25932	28754/5.45	9	123	44	28	2D
**3**	Q05823	2-5A-dependent ribonuclease (Ribonuclease L)	mRNA processing and protein phosphorylation	RN5A_HUMAN	RNASEL	6041	83481/6.20	13	33	20	54	2D
**4**	P49368	T-complex protein 1, gamma subunit	Protein folding	TCPG_HUMAN	CCT3	7203	60364/6.10	12	56	22	24	2D
**5**	P68104	EF-1-alpha-1	Translation elongatrion	EF1A1_HUMAN	EEF1A1	1915	50109/9.10	9	80	22	15	2D
**6**	P09104	Gamma enolase	Glycolysis	ENOG_HUMAN	ENO2	2026	47108/4.91	8	98	27	22	2D
**7**	O00629	Importin alpha-4 subunit	Protein trasport	IMA4_HUMAN	KPNA4	3804	57851/4.80	9	142	22	21	2D
**8**	P06748	Nucleophosmin (NPM)	Anti-apoptotic function	NPM_HUMAN	NPM1	4869	32555/4.64	6	149	28	50	2D: IB
**9**	P30101	Protein disulfide-isomerase A3 precursor	Redox homeostasis and signal transduction	PDIA3_HUMAN	PDIA3	2923	56747/5.98	15	146	30	34	2D
**10**	P14618	Pyruvate kinase	Gycoloysis & programmed cell death	KPYM_HUMAN	PKM2	5315	57769/7.95	15	121	35	51	2D
**11**	Q12765	Secernin 1	Exocytosis and proteolysis	SCRN1_HUMAN	SCRN1	9805	46353/4.66	12	60	33	61	2D
**12**	P02768	Serum albumin precursor	Starvation response and anti-apoptotic	ALBU_HUMAN	GLB	213	69321/5.92	9	145	12	61	2D
**13**	P30049	ATP synthase delta chain	ATP Catabolism	ATPD_HUMAN	ATP5D	513	17479/5.38	3	85	17	25	2D
**14**	P43686	26S protease regulatory subunit 6B	Protein catabolic process	PRS6B_HUMAN	PSMC4	5704	47337/5.09	11	81	26	25	2D
**15**	P35998	26S protease regulatory subunit 7	Protein catabolic process	PRS7_HUMAN	PSMC2	5701	48472/5.72	23	367	52	34	2D
**16**	Q92878	DNA repair protein RAD50 (hRAD50)	DNA Unwinding	RAD50_HUMAN	RAD50	10111	153797/6.48	22	31	16	73	2D: IB
**17**	P49411	Elongation factor Tu, (EF-Tu) (P43)	Translational elongation	EFTU_HUMAN	TUFM	7284	49510/7.26	15	238	34	47	2D
**18**	Q02790	FK506-binding protein 4 (PPIase)	Protein folding	FKBP4_HUMAN	FKBP4	2288	51641/5.35	9	99	23	46	2D
**19**	P10809	Hsp60	Protein stablization	CH60_HUMAN	HSPD1	3329	61016/5.70	24	708	45	28	2D
**20**	P32119	Peroxiredoxin 2	Anti-apoptosis and maintenance of redox homeostasis	PRDX2_HUMAN	PRDX2	7001	21878/5.66	11	390	40	23	2D
**21**	P13489	Placental ribonuclease inhibitor (RAI)	mRNA catabolism & angiogenesis regulation	RINI_HUMAN	RNH1	6050	49810/5.71	17	730	75	29	2D
**22**	P31943	Heterogeneous nuclear ribonucleoprotein H	mRNA processing and regulation	HNRH1_HUMAN	HNRNPH1	3187	49067/5.89	14	92	38	18	2D
**23**	P06576	ATP synthase beta chain, mitochondrial precursor	Angiogenesis & ATP synthesis	ATPB_HUMAN	ATP5B	506	56525/5.26	15	81	37	27	2D
**24**	P45973	Chromobox protein homolog 5	Chromatin assembly and disassembly	CBX5_HUMAN	CBX5	23468	22211/5.71	10	144	60	24	2D
**25**	P51571	Translocon-associated protein, delta subunit precursor	Intracellular protein transport	SSRD_HUMAN	SSR4	6748	18987/5.76	5	236	36	16	2D
**26**	P00441	Superoxide dismutase [Cu-Zn]	Apoptosis and cellular maintenance	SODC_HUMAN	SOD1	6647	15795/5.70	3	62	29	11	2D
**27**	P61326	Mago nashi protein homolog	mRNA processing	MGN_HUMAN	MAGOH	4116	17153/5.74	9	280	54	17	2D
**28**	O94832	Myosin Id	Cellular dynamics	MYO1D_HUMAN	MYOD1	4642	116129/9.44	19	46	15	52	2D
**29**	P62306	Small nuclear ribonucleoprotein F	Spliceosomal snRNP assembly	RUXF_HUMAN	SNRPF	6636	9719/4.70	5	226	33	31	2D
**30**	P49720	Proteasome subunit beta type 3	Protein catabolism	PSB3_HUMAN	PSMB3	5691	22933/6.14	8	96	49	55	2D
**31**	P61769	Beta-2-microglobulin precursor	Antigen processing and immune response	B2MG_HUMAN	B2M	567	13706/6.06	2	44	13	16	2D
**32**	Q13011	Delta3,5-delta2,4-dienoyl-CoA isomerase, mitochondrial precursor	Fatty acid beta oxidation	ECH1_HUMAN	ECH1	1891	35971/6.61	6	76	15	25	2D
**33**	P25786	Proteasome subunit alpha type 1 Macropain subunit	Protein catabolism and immune response	PSA1_HUMAN	PSMA1	5682	29537/6.15	7	119	29	16	2D
**34**	P50990	T-complex protein 1, theta	Protein folding	TCPQ_HUMAN	CCT8	10694	59451.5	7	46	15	52	2D

**Table 2:** Details of proteins identified through MALDI-TOF (MS/MS) analysis in Group II.

Sub-group I. Proteins differentially up-regulated in A4-P cells.													
Spot No.	Accession No.	Description of identified proteins	Function	SwissProt Accession	Gene name	Gene ID	Mass (Da)/PI	Peptide matched	Score	Sequence coverage [%]	RMS (ppm)	Fold change (A4-P/T)	Validation method
**1**	P84103	Splicing factor, arginine/serine-rich 3	RNA splicing and processing	SFRS3_HUMAN	SFRS3	6428	19318/11.64	9	153	54	48	2.608228	2D: IB
**2**	O94925	Glutaminase	glutamine catabolic process	GLS_HUMAN	GLS	2744	73414/7.85	15	540	36	19	2.103207	2D
**3**	P31937	3-hydroxyisobutyrate dehydrogenase, mitochondrial	oxidation reduction	HIBADH_HUMAN	HIBADH	11112	35306/8.38	8	199	27	33	2.88171	2D
**4**	P08754	Guanine nucleotide-binding ptn G(k),alpha	negative regulation of adenylate cyclase activity	GNAI3_HUMAN	GNAI3	2773	40375/5.51	14	166	36	30	4.086129	2D
**5**	P62873	Guanine nucleotide-binding protein G(I)/G(S)/G(T) subunit beta-1	Ras protein signal transduction/hormone signaling	GNB1L_HUMAN	GNB1L	2782	37353/5.6	13	311	44	24	2.789543	2D
**6**	P31930	Ubiquinol cytochrome C reductase complex core ptn I	aerobic respiration/proteolysis/transport	UQCRC1_HUMAN	UQCRC1	7384	52585/5.94	20	592	53	16	3.247917	2D
**7**	P28331	NADH ubiquinone oxidoreductase subunit	ATP metabolic process/apoptosis/transport	NDUFS1_HUMAN	NDUFS1	4719	79465/5.89	21	249	42	35	13.41311	2D: IB
**8**	O43707	Alpha actinin-4	cellular component movement/protein transport/regulation of apoptosius	ACTN4_HUMAN	ACTN4	81	104788/5.27	31	462	37	38	2.19378	2D
**9**	Q07955	Splicing factor, arginine/serine-rich 1	mRNA splice site selection	SFRS1_HUMAN	SFRS1	6426	27597/10.37	6	172	43	29	6.715184	2D: IB
**10**	Q14974	Importin β subunit	NLS-bearing substrate import into nucleus/protein import & translocation	IMB1_HUMAN	IMB1	3837	97108/4.68	30	926	50	11	11.88048	2D: IB
**11**	Q86UE8	Serine/threonine-protein kinase tousled-like 2	intracellular signaling pathway/chromatin modification	TLK2_HUMAN	TLK2	11011	80606/8.65	17	45	15	73	6.609533	2D
**12**	P68363	Tubulin alpha-ubiquitous chain	microtubule-based movement/protein polymerization	TUBA1B_HUMAN	TUBA1B	10376	50120/4.94	11	88	33	28	4.449974	2D
**13**	P20700	Lamin B1	protein binding/structural molecule activity	LMNB1_HUMAN	LMNB1	4001	66237/5.11	20	220	37	26	2.990194	2D
**14**	P61978	Heterogeneous nuclear ribonucleoprotein K (hnRNP K)	RNA splicing/mRNA processing/signal transduction	HNRNPK_HUMAN	HNRNPK	3190	50944/5.39	18	425	40	37	2.466555	2D
**15**	P11021	78 kDa glucose-regulated protein	ER-associated protein catabolic process/anti-apoptosis	HSPA5_HUMAN	HSPA5	3309	72288/5.07	14	753	52	18	26.707	2D
**16**	O75116	Rho-associated protein kinase 2	cytokinesis/protein amino acid phosphorylation	ROCK2_HUMAN	ROCK2	9475	160812/5.75	31	73	18	50	7.122232	2D
**17**	P22314	Ubiquitin-activating enzyme E1 (A1S9 protein)	cell death/protein modification process	UBA1_HUMAN	UBA1	7317	117774/5.49	24	339	25	20	2.52764	2D
**18**	P05388	60S acidic ribosomal protein P0 (L10E)	ribosome biogenesis/translational elongation	RPLP0_HUMAN	RPLP0	6175	34252/5.71	12	287	43	23	2.007637	2D
**19**	P62140	Serine/threonine protein phosphatase PP1-beta catalytic subunit (PP-1B)	cell cycle/cell division/glycogen metabolic process	PPP1C_HUMAN	PPP1CB	5500	37163/5.84	7	91	19	45	16.02527	2D
**20**	Q99497	DJ-1 protein (Oncogene DJ1)	cell death/regulation of androgen receptor signaling pathway	PARK7_HUMAN	PARK7	11315	19878/6.33	8	221	53	18	2.102286	2D
**21**	P50395	Rab GDP dissociation inhibitor beta (Rab GDI beta) (GDI-2)	protein transport/regulation of GTPase activity/signal transduction	GDI2_HUMAN	GDI2	2665	50631/6.11	18	324	44	31	2.027135	2D
**22**	P24534	Elongation factor 1-beta (EF-1-beta)	translational elongation	EEF1B2_HUMAN	EEF1B2	1933	24617/4.50	9	329	52	6	2.678404	2D
**23**	P67936	Tropomyosin alpha 4 chain (Tropomyosin 4)	cellular component movement/muscle contraction	TPM4_HUMAN	TPM4	7171	28373/4.67	17	507	51	23	2.033974	2D
**24**	Q9Y297	F-box/WD-repeat protein 1A	Wnt receptor signaling pathway/ubiquitin-dependent protein catabolic process	BTRC_HUMAN	BTRC	8945	68822/8.30	17	46	26	50	2.223773	2D
**25**	Q92973	Transportin 1 (Importin beta-)2	interspecies interaction between organisms/protein import into nucleus	TNPO1_HUMAN	TNPO1	3842	101244/4.81	15	209	17	12	4.892821	2D
**26**	P06733	Alpha enolase 2-phospho-D-glycerate hydro-lyase)	glycolysis/negative regulation of cell growth	ENO1_HUMAN	ENO1	2023	47008/6.99	15	330	42	73	2.858116	2D
**27**	P13645	Cytokeratin 10	epidermis development	KRT10_HUMAN	KRT10	3858	59483/5.13	18	281	32	15	141.9166	2D: IB
**28**	Q03252	Lamin B2	Structural molecule activity	LMNB2_HUMAN	LMNB2	84823	67647/5.29	30	603	51	22	7.605702	2D
**29**	Q9NR28	Diablo homolog, Mitochonria-deriver caspase cativator	Activation of caspase activity by Cyt C/ Induction of appoptosis	DIABLO_HUMAN	DIABLO	56616	27114/5.68	11	140	39	28	4.085068	2D: IB
**30**	P08670	Vimentin	cellular component movement	VIM_HUMAN	VIM	7431	53488/5.06	32	939	42	73	3.239119	2D: IB
**31**	P50224	Monoamine-sulfating phenol sulfotransferase	catecholamine metabolic process, steroid metabolic process	SULT1A3_HUMAN	SULT1A3	4E+05	34174/5.68	12	124	49	31	6.744596	2D

Sub-group II. Proteins differentially upregulated in A4-T cells.													
Spot no	Accession no.	Description of identified proteins	Function	SwissProt Accession	Gene name	Gene ID	Mass (Da)/PI	Peptide matched	Score	Sequence coverage [%]	RMS (ppm)	Fold change (A4-T/P)	Validation method
**1**	P23526	Adenosylhomocysteinase	one-carbon metabolic process	AHCY_HUMAN	AHCY	191	47554/5.92	16	223	33	29	2.828966	2D
**2**	P05787	Cytokeratin 8	cytoskeleton organization	KRT8_HUMAN	KRT8	3856	53510/5.52	33	530	53	16	2.141424	2D: IB
**3**	P63261	Gamma actin	cellular component movement	ACTG1_HUMAN	ACTG1	71	41766/5.31	15	417	49	30	4.030835	2D: IB
**4**	Q15084	Protein disulfide isomerase A6	cell redox homeostasis/protein folding	PDIA6_HUMAN	PDIA6	10130	48091/4.95	12	357	35	8	8.528689	2D
**5**	P07900	HSP 90 Alpha	cellular chaperone-mediated protein complex assembly	HSP90AA1_HUMAN	HSP90AA1	3320	84490/4.94	12	60	18	50	2.561739	2D
**6**	P52788	Spermine synthase	methionine metabolic process/spermine biosynthetic process	SMS_HUMAN	SMS	6611	41242/4.87	9	68	23	26	2.087467	2D
**7**	O00410	Importin subunit-3	NLS-bearing substrate import into nucleus	IPO5_HUMAN	IPO5	3843	123550/4.83	21	251	23	19	2.005113	2D
**8**	P14625	Endoplasmin precursor (GRP94)	ER-associated protein catabolic process/anti-apoptosis	ENPL_HUMAN	ENPL	7184	92411/4.76	36	609	47	7	56.43346	2D
**9**	Q14764	Major vault protein (MVP)	mRNA and protein transport	MVP_HUMAN	MVP	9961	99135/5.34	23	214	28	20	7.717112	2D
**10**	P08238	Heat shock protein HSP 90-beta	protein folding	HSP90AB1_HUMAN	HSP90AB1	3326	83081/4.97	16	64	17	40	5.908433	2D
**11**	Q99426	Tubulin-specific chaperone B	cell differentiation/nervous system development	TBCB_HUMAN	TBCB	1155	27308/5.06	9	79	33	45	3.162533	2D
**12**	Q9BT78	COP9 signalosome complex subunit 4	protein binding	COPS4_HUMAN	COPS4	51138	46240/5.57	15	207	37	17	2.618724	2D
**13**	O15372	eIF-3 gamma (eIF3 p40 subunit)	regulation of translational initiation	EIF3H_HUMAN	EIF3H	8667	39905/6.09	13	71	28	37	3.177448	2D
**14**	P29692	Elongation factor 1-delta (EF-1-delta)	positive regulation of I-kappaB kinase/NF-kappaB cascade/translational elongation	EEF1D_HUMAN	EEF1D	1936	30972/4.90	11	204	43	5	4.075992	2D
**15**	Q15366	Poly(rC)-binding protein 2 (Alpha-CP2)	RNA splicing/mRNA metabolic process	PCBP2_HUMAN	PCBP2	5094	38556/6.33	10	72	35	41	4.952897	2D
**16**	P28070	Proteasome subunit beta type 4 precursor	anaphase-promoting complex-dependent proteasomal ubiquitin-dependent protein catabolic process	PSMB4_HUMAN	PSMB4	5692	29173/5.72	7	96	27	28	3.783769	2D
**17**	P27348	14-3-3 protein tau (14–3–3 protein theta)	negative regulation of transcription, DNA-dependent	YWHAQ_HUMAN	YWHAQ	10971	27747/4.68	15	216	55	18	2.880806	2D
**18**	Q13952	Nuclear factor NF-Y protein chain C	protein folding/regulation of transcription	NFYC_HUMAN	NFYC	4802	50271/5.78	3	66	7	15	4.573116	2D
**19**	P60228	Eukaryotic tanslation initiation factor 3 subunit eIF-3 p48	translational initiation/nuclear-transcribed mRNA catabolic process	EIF36_HUMAN	EIF36	3646	52187/5.71	11	49	24	36	8.501138	2D
**20**	P13693	Translationally controlled tumor ptn(TCTP)(p23)	anti-apoptosis/cellular calcium ion homeostasis	TPT1_HUMAN	TPT1	7178	19583/4.84	7	70	33	20	3.649924	2D
**21**	P08779	Cytokeratin 16	cell proliferation/cytoskeleton organization	KRT16_HUMAN	KRT16	3868	51105/4.98	11	101	24	26	2.869215	2D
**22**	P04792	HspB1Heat shock 27 kDa protein	anti-apoptosis/cellular component movement/regulation of translational initiation	HSPB1_HUMAN	HSPB1	3315	22768/5.98	10	163	46	11	3.099848	2D
**23**	P60842	Eukaryotic initiation factor 4A-I (eIF4A-I)	translation	EIF4A1_HUMAN	EIF4A1	1973	46125/5.32	21	574	45	40	6.608386	2D
**24**	P17987	T-complex protein 1, alpha (TCP-1-alpha)	protein folding/tubulin complex assembly	TCP1_HUMAN	TCP1	6950	60306/5.80	20	144	38	22	3.35298	2D
**25**	P62258	14–3–3 protein epsilon	apoptosis/induction of apoptosis by extracellular signals	YWHAE_HUMAN	YWHAE	7531	29155/4.63	16	257	61	30	2.060479	2D
**26**	P12004	Proliferating cell nuclear antigen (PCNA) (Cyclin)	DNA replication/cell proliferation/mismatch repair	PCNA_HUMAN	PCNA	5111	28750/4.57	11	519	52	21	3.664895	2D
**27**	Q01105	SET protein	DNA replication/nucleocytoplasmic transport	SET_HUMAN	SET	6418	33469/4.23	10	491	38	25	3.358717	2D
**28**	P27797	Calreticulin precursor ERp60	cell cycle arrest/protein stabilization/regulation of apoptosis	CALR_HUMAN	CALR	811	48112/4.29	14	1180	49	35	11.82921	2D
**29**	P28072	Proteasome subunit beta type 6 precursor (Proteasome delta chain)	anaphase-promoting complex-dependent proteasomal ubiquitin-dependent protein catabolic process	PSMB6_HUMAN	PSMB6	5694	25341/4.80	7	166	30	15	2.770366	2D
**30**	P08727	Cytokeratin 19	response to estrogen stimulus/sarcomere organization	KRT19_HUMAN	KRT19	3880	44079/5.04	30	861	69	20	2.032384	2D: IB
**31**	O94905	Protein C8orf2	ER-associated protein catabolic process	ERLIN2_HUMAN	ERLIN2	11160	37815/5.47	9	124	27	40	4.069573	2D
**32**	P14923	Junction plakoglobin (Desmoplakin III)	cell migration/endothelial cell-cell adhesion/detection of mechanical stimulus	JUP_HUMAN	JUP	3728	81447/5.95	19	230	30	45	2.078487	2D
**33**	P23381	Tryptophanyl-tRNA synthetase (Tryptophan--tRNA ligase)	regulation of angiogenesis/tryptophanyl-tRNA aminoacylation	WARS_HUMAN	WARS	7453	53132/5.83	10	87	20	47	2.423008	2D
**34**	P15311	Ezrin (p81)	actin filament bundle assembly/membrane to membrane docking	EZR_HUMAN	EZR	7430	69225/5.95	12	510	54	32	3.924348	2D: IB
**35**	Q9Y230	RuvB-like 2 (48-kDa TATA box-binding protein-interacting protein)	DNA recombination & recombination/regulation of transcription	RUVBL2_HUMAN	RUVBL2	10856	51125/5.49	22	292	47	42	4.228731	2D
**36**	P51570	Galactokinase (Galactose kinase)	carbohydrate phosphorylation/galactose metabolic process	GALK1_HUMAN	GALK1	2584	42246/6.04	14	129	34	37	2.024403	2D
**37**	P04181	Ornithine aminotransferase, mitochondrial precursor	visual perception	OAT_HUMAN	OAT	4942	48504/6.57	16	223	41	29	3.552906	2D
**38**	Q10713	Mitochondrial processing peptidase alpha subunit, precursor	proteolysis	PMPCA_HUMAN	PMPCA	23203	58216/6.45	14	106	27	82	33.28102	2D
**39**	Q9Y265	RuvB-like 1 (49-kDa TATA box-binding protein-interacting protein)	DNA recombination/cell division/regulation of growth	RUVBL1_HUMAN	RUVBL1	8607	50196/6.02	15	135	30	76	11.4513	2D
**40**	P04083	Annexin A1	anti-apoptosis/cellular component movement/cell surface receptor linked signaling pathway	ANXA1_HUMAN	ANXA1	301	38559/6.64	16	487	57	65	12.0573	2D: IB
**41**	P07237	Protein disulfide-isomerase precursor (PDI)	cell redox homeostasis,	P4HB_HUMAN	P4HB	5034	57081/4.76	17	399	30	40	2.336195	2D
**42**	P07910	Heterogeneous nuclear ribonucleoproteins C1/C2 (hnRNP C1/hnRNP C2)	nuclear mRNA splicing, via spliceosome	HNRNPC_HUMAN	HNRNPC	3183	33667/4.95	12	246	35	47	2.011891	2D
**43**	P17655	Calpain-2 subunit II precursor	proteolysis	CAPN2_HUMAN	CAPN2	824	79828/4.88	13	121	24	15	19.41388	2D
**44**	P08107	Heat shock 70 kDa protein 1	Stress response	HSPA1A_HUMAN	HSPA1A	3303	70009/5.48	26	676	40	22	2.015034	2D: IB
**45**	P38646	Stress-70 protein (75 kDa glucose regulated protein) (GRP 75)	protein import into nucleus, translocation	GRP75_HUMAN	GRP75	3842	73635/5.87	23	250	34	27	2.112865	2D: IB
**46**	P30048	Thioredoxin-dependent peroxide reductase, mitochondrial precursor	cell redox homeostasis/regulation of mitochondrial membrane potential	PRDX3_HUMAN	PRDX3	10935	27675/7.67	7	210	37	17	2.91228	2D
**47**	Q9BS26	Thioredoxin domain-containing protein 4	cell redox homeostasis	TXND4_HUMAN	ERP44	23071	46941.4/5.09	15	441	39	38	4.879818	2D
**48**	P05783	Cytokeratin 18	protein binding, structural molecule activity	KRT18_HUMAN	KRT18	3875	53488/5.34	16	627	43	27	4.928731	2D: IB

### Categorization of functional pathways based on reported ontologies and expression validation

Gene ontology analyses further identified distinct molecular functionalities and pathways associated with the identified protein profiles in the two cell types (Fig. 1D.i). Thus, several cellular regulatory mechanisms including protein biosynthesis, cytoskeleton organization, signal transduction, regulation of apoptosis and protein degradation were largely enriched in and contributed to the functionality of A4-P cells. These were suggested to have a cross-talk with other pathways such as protein and energy metabolism, RNA metabolism, cellular differentiation and redox reactions.

Conversely, functional grouping of the proteins in A4-T cells comprised pathways associated with the classical hallmarks of cancer cells *viz*. resistance to apoptosis, energy metabolism, cell proliferation, angiogenesis and invasion and metastases (Fig. 1D.ii). Thus, molecules involved in associated with protein biosynthesis, folding and transport control the dynamic process of protein metabolism in transformed cells towards matching its proliferative activities.

**Figure 2:**
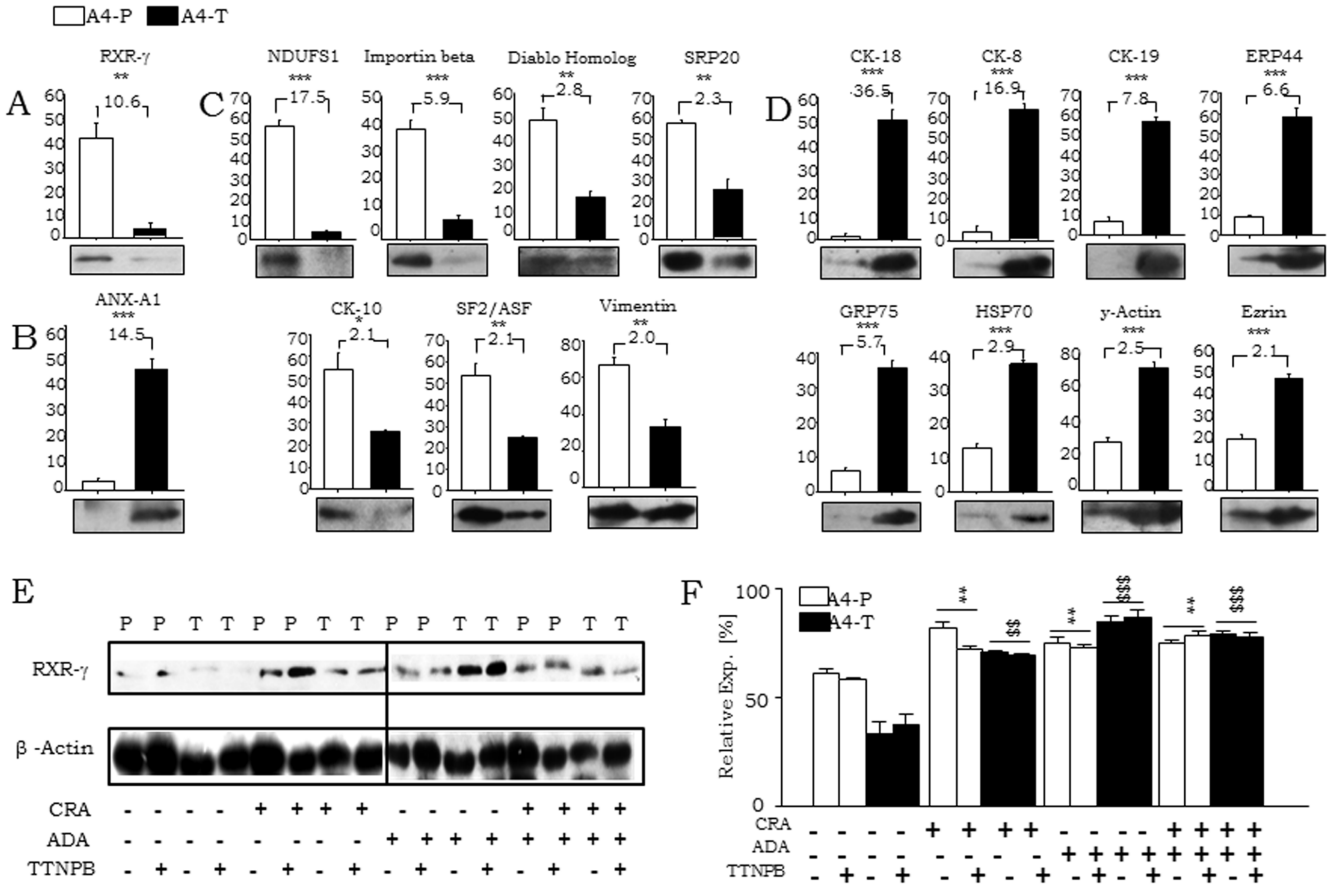
Validation of differentially expressed proteins and induction of RXR-γ levels on retinoid treatments. Quantitative validation of the expression and fold change of some proteins, identified in both groups; Group I proteins, A, exclusively expressed in A4-P and B, in A4-T cell respectively; whereas D, group-II proteins quantitatively up-regulated in A4-P cells and E, in A4-T. E. Relative expression of RXR-γ and β-actin in CRA, ADA or TTNPB retinoids treated A4-P (P) and A4-T (T) cells validated through immunoblotting. F. Quantitation of relative RXR-γ expression in A4-P and A4-T cells. Statistical analysis showing test of significance (*-control A4-P and retinoids treated cells; $- control A4-P and retinoids treated cells).The data shown are representative of three separate experiments and depicted as mean ± SEM *p<0.05, **p<0.01, ***p<0.001.

Towards confirming levels of some of the identified proteins, their expressions were validated between A4-P and A4-T cells through immunoblotting (Fig. 1E; Fig. 2A,B,C,D). SeOvCa is unique in that, transformation is associated with expression of epithelial markers [21]. Enhanced vimentin expression in A4-P cells suggest mesenchymal while elevated levels of Cytokeratin 8 and 18 in A4-T cells correlate with epithelial features respectively (Fig. 1E), besides being in concordance with their cell morphology and the prevalent hypothesis.

### Functional characterization of RXR-γ an exclusive Group I protein

Ten Group I proteins were expressed exclusively in the A4-P cells (EEx proteins). Literature-based functional annotation led to their categorizat ion into four functional groups *viz.*

- *Cell differentiation and apoptosis –* RXR-γ, PRDX4;
- *Cell proliferation* – GNA11, PIBF-1, ANXA5, Cathepsin D (CTSD);
- *Mitosis and cytokinesis* – KLH9;
- *Epithelial-mesenchymal transition (EMT)* – TPM1, ANXA5.

While all the above functions are relevant in the process of transformation, we focused on studying the functionality of cellular differentiation and apoptosis that is critical in maintaining tissue homeostasis and known to be regulated by RXR-γ at the transcriptional level by dimerizing with retinoic acid or retinoic acid X receptors (RAR or RXR respectively) or other permissive heterodimer partners like PPAR-γ [22], [23], [24]. We thus decided to investigate the role of RXR-γ in our epithelial ovarian cancer progression model.

### RXR-γ interactions with nuclear receptors and modulation of cellular differentiation in A4-P cells upon retinoids treatment

**Figure 3:**
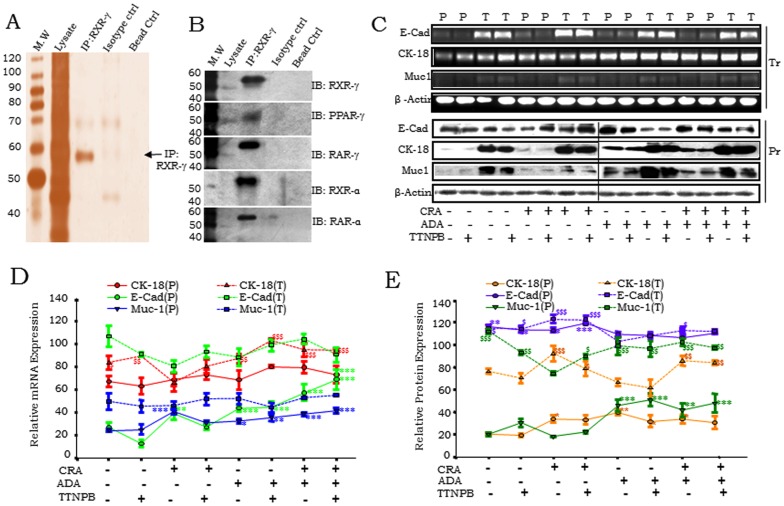
RXR-γ interacts with a number of nuclear receptors and modulates cellular differentiation. A. Co-Immunoprecipitation (Co-IP) with RXR-γ showing eluted Immunocomplex by silver staining. B. Validation of RXR-γ indicating interaction in Co-IP with RXR-γ with PPAR-γ, RAR-γ, RXR-α and RAR-α in A4-P cells validated through immunoblotting. C. Expression profiling of CK-18, Muc-1 and E-Cadherin at transcriptional (Tr) and protein (Pr) performed by semi-quantitative RT-PCR and immunoblotting in CRA, ADA or TTNPB retinoids treated A4-P (P) and A4-T (T) cells. D. Quantitation of mRNA expression of E-Cad, CK18 and MUC1 epithelial differentiation markers in A4-P (P; line) and A4-T cells (T; dashed line) upon retinoids treatment validated through RT-PCR. E. Quantitation of protein expression of E-Cad, CK18 and MUC1 makers in A4-P (P; line) and A4-T cells (T; dashed line) upon retinoids treatment validated through immunoblotting. Data shown are representative of three separate experiments depicted as mean ± SEM *p<0.05, **p<0.01, ***p<0.001.

Retinoid treatment enhanced RXR-γ levels in A4-P cells; and interestingly, resumed significant expression in A4-T cells as well (Fig. 2E,F). CRA and ADA individual treatment elevated RXR-γ levels in both cell types, though this induction was less effective in combination with TTNPB. Towards validation of RXR-γ interactions with other nuclear receptors, co-immunoprecipitation and immunoblotting affirmed interactions with PPAR-γ, RAR-γ, RXR-α and RAR-α in pre-transformed cells (Fig. 3A,B). Evaluation of RXR-γ involvement in cellular differentiation was achieved through profiling epithelial markers E-cadherin (E-cad), Cytokeratin 18 (CK-18) and Mucin-1 (Muc-1) at gene expression and protein levels, in steady state and on exposure to natural *viz*. CRA and synthetic retinoids (ADA and TTNPB) (Fig. 3C). At steady state, lower expression of E-Cad was observed in A4-P cells. Expression of E-Cad further increased with CRA and also with ADA; CRA was given alone or in combination with ADA and TTNPB. Levels of E-Cad, CK18 and Muc-1 were endogenously higher in A4-T cells. Synthetic retinoid ADA alone or in combination with CRA upregulated CK18 expression in both cell types. Although, specific role of TTNPB in cellular differentiation is unknown, TTNPB treatment resulted in minor upregulation of differentiation markers. All three markers were enhanced in response to retinoid exposure in A4-P cells thereby affirming the involvement of RXR-γ in modulation of cellular differentiation. Retinoid treatment in the A4-T cells resulted in induction of RXR-γ without any significant alterations in the levels of these epithelial differentiation marker at gene expression and protein levels (Figs. 3D,3E).

### Retinoid induced RXR-γ levels sensitize transformed cells towards apoptosis via intrinsic pathway

**Figure 4:**
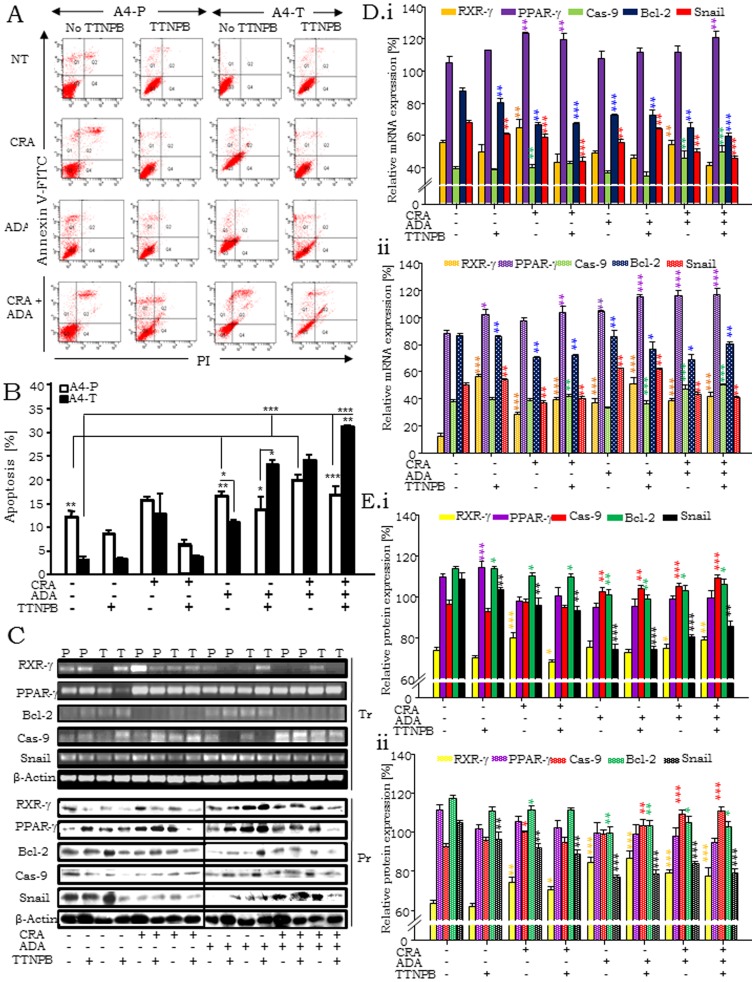
RXR-γ levels sensitize cellular apoptosis in A4-T cells upon retinoid treatment. A. Annexin V-FITC assay data showing apoptosis in A4-P and A4-T cells on different retinoids treatment regimes; where i. having no retinoid treatment, ii. treated with CRA, iii. with ADA and iv. with both having alternative treatment of another synthetic retinoid i.e. TTNPB. B. Statistical analysis of apoptosis assay showing significant apoptosis among both cell types in different sets of retinoid treatment. C. Expression analyses of RXR-γ, PPAR-γ, Bcl-2, Caspase 9 and snail at transcriptional (Tr.) and protein level (Pr.) through RT-PCR and immunoblotting respectively. D. Quantitation of mRNA expression, i. expression of RXR-γ, PPAR-γ, Caspase 9, Bcl-2 and snail upon retinoids treatment in A4-P cells whereas, ii. shows their levels in A4-T cells on validation through RT-PCR. E. Quantitation of protein expression, i. expressions of RXR-γ, PPAR-γ, Caspase 9, Bcl-2 and snail in A4-P cells whereas, ii. shows their levels in A4-T cells upon retinoids treatment validated through immunoblotting.

Role of RXR-γ in mediating apoptosis in response to natural and synthetic retinoids was evaluated (Fig. 4A). At steady state, apoptosis was significantly lower in A4-T cells as compared to A4-P cells indicating acquisition of resistance to apoptosis during the transformation process. Apoptosis was enhanced in both cell types on exposure to CRA and ADA – either alone or in combination (Fig.4B). While TTNPB by itself failed to induce cell death, in combination with ADA and CRA it sensitized A4-T cells to apoptosis. Retinoid mediated activation of RXR-γ expression was also found to correlate directly with higher levels of apoptosis (Fig. 2E,F). We profiled expression of the transcription factor Snail (that antagonizes p53-mediated pro-survival signaling through active repression of the pro-apoptotic molecules PUMA/BBC3, ATM and PTEN in ovarian cancer cells under stress; [19]) to evaluate the effect of RXR-γ led apoptosis on it. Caspase 9, a marker of intrinsic apoptosis pathway, upregulated during RXR-γ and PPAR-γ induction and Bcl-2 as markers of apoptosis [25]. Snail and Bcl-2 expression were reduced, while significantly elevated expression of RXR-γ, PPAR-γ and Caspase 9 were evident on retinoid treatment (Fig. 4C, 4D, 4E). The expression of these molecules was further enhanced on combinatorial retinoid treatment. We further probed the effects of retinoids on cell cycle profiles (Fig. S1A,1B). As expected, steady state A4-T cells possess higher S & G2/M populations than A4-P cells indicating active proliferation. CRA treatment enhances apoptosis along with G2/M arrest in A4-P cells while ADA and TTNPB induce only G2/M arrest. In the combinatorial treatments, CRA with ADA or presence of all three retinoids induced a G1/S arrest in transformed cells.

### In vivo retinoid treatment significantly reduce xenograft growth in NOD-SCID mice through RXR-γ mediated sensitization of transformed cells towards apoptosis

**Figure 5:**
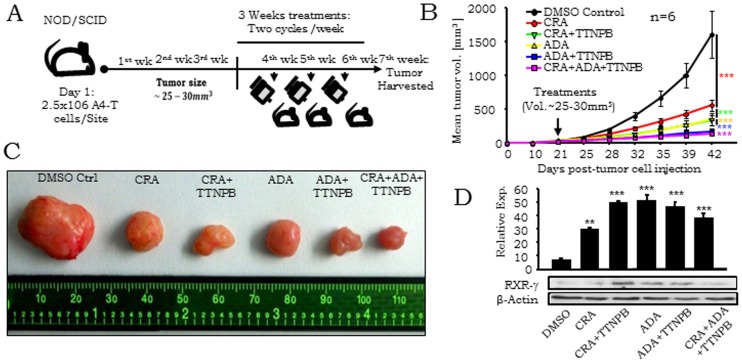
*In vivo* retinoid treatment resumes RXR-γ levels and reduces xenograft growth. A. Experimental procedure illustrating retinoids treatment regime in NOD-SCID mice. Mice were observed upto 3 week until tumor size grows 25–30 mm^3^, treatment of DMSO, CRA, CRA+TTNPB, ADA, ADA+TTNBP and CRA+ADA+TTNBP started on 4^th^ week and proceeded upto 7^th^ week. B. Graphical representation showing tumor volumes of control and retinoids treated NOD-SCID mice at different time points. C. Comparative tumor sizes of control and retinoids treated tumors. D. Quantitative expression of RXR-γ in control and retinoids treated tumors validated through immunoblotting. Data shown are representative of three separate experiments (n = 6 for *in vivo* experiments) and depicted as mean ± SEM *p<0.05, **p<0.01, ***p<0.001.

We further extended the above re-sensitization of RXR-γ levels in A4-T cells effects obtained *in vitro* to experimental tumors (Fig. 5A). Mean tumor volume (Figs. 5B,5C) at each treatment point along with mean tumor volume and weight at 7th week (Figs. S1C, S1D) showed significant reduction in retinoid treated mice tumors vs. those in DMSO treated controls. Overall, the combinatorial retinoid treatment was most effective. The distinctly upregulated RXR-γ expressions in the retinoid-treated tumors strongly suggest sensitization of transformed A4 cells to apoptosis. Combined effect of retinoids was found to be most lethal for tumor growth through resumed RXR-γ mediated apoptosis of tumor cells *in vivo*. RXR-γ levels were found significantly higher in all 5 sets including CRA, CRA & TTNPB, ADA, ADA & TTNPB and CRA, ADA & TTNPB; in comparison to DMSO vehicle control. This is a definitive correlation with RXR-γ stimulation and induction of apoptosis in these cells *in vitro* (Fig.5D).

## Discussion

The existence of several histological sub-types that correlate with different cell(s)-of-origin in ovarian cancer [26] remains a hurdle in the establishment of representative progression models in this disease. This is in contrast to other malignancies such as prostate cancer in which such models have been applied over the last two decades in elucidating molecular mechanisms of disease [1], [27], [28], [29]. In the present study, detailed exclusive and differential protein profiling of a progression model established earlier in our lab provided novel insights into altered molecular patterns during SeOvCa progression. A4-P cells with replicative immortality represent a pre-neoplastic stage while A4-T cells with aggressive and metastatic characteristics are representative of transformation and disease progression.

**Figure 6:**
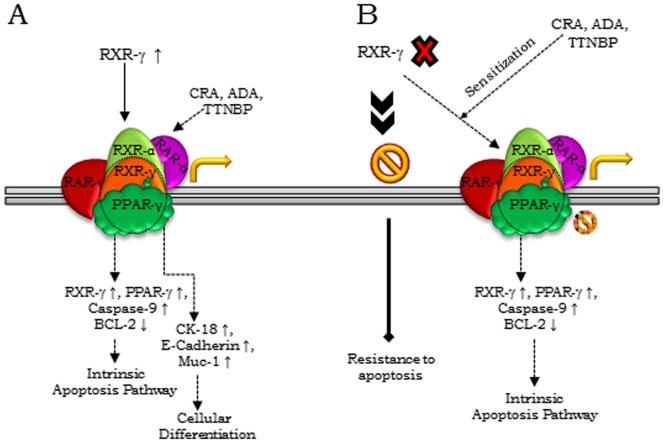
Schematic model showing modulation of cellular differentiation and apoptosis by RXR-γ during the progression of epithelial ovarian cancer. A. RXR-γ modulation at steady state in pre-transformed cells; retinoids treatment enhances RXR-γ levels and scale up apoptosis (upon RXR-γ interaction with PPAR-γ) and expression of epithelial differentiation specific markers (upon RXR-γ interactions with RAR-γ, RXR-α and RAR-α). B. Deficiency of RXR-γ providing benefits of resistance to apoptosis to transformed cells; retinoid treatment induced RXR-γ levels sensitize these cells towards significant apoptosis.

Our data affirms that the two functional states of the model are associated with distinct protein profiles. Within the group of proteins exclusive to the A4-P cells, characterization of the role of RXR-γ revealed a sensitivity of the pre-transformed cells to apoptosis and differentiation as described earlier [30]. Compromised RXR-γ levels are also reported in several malignancies including non-small cell lung cancer [31]; where it also has been reported that epigenetic silencing of RXR-γ correlated with decreased overall survival of patients [32]. In our pre-transformed cells, RXR-γ cooperates with PPAR-γ, RAR-γ, RAR-α and RXR-α to form functional heterodimeric complexes, where RXR-γ with PPAR-γ coordinates cellular apoptosis through the intrinsic pathway confirmed with elevated Caspase-9 levels. Further, we observed that RXRγ activation in transformed cells re-sensitizes them to apoptosis as a synergistic effect of agonists that mediate cytotoxic effects *in vitro* as well as in experimental tumors (Fig. 6). This is an important identification towards application of retinoid-based therapies. In this study, we characterized the pleotropic nature of RXR-γ signaling in our SeOvCa-progression model system. Loss of RXR-γ levels indicated to facilitate mechanistic benefits to transformed cells towards acquisition of resistance to apoptosis; consequently, retinoid-sensitized tumor cells upregulate RXR-γ levels leading to significant cell death.

The present proteomics approach is a first account of changes in SeOvCa that reflect on various transformation–associated functional pathways. Significantly, RXR-γ signaling could be a potential gateway in preventing disease progression. The elucidation of RXR-γ signaling extends contemporary approaches of cellular transformation in SeOvCa that can now be exploited further in development and evaluation of new therapeutic modalities.

## Material and Methods

### Ethics statement

All animal work was conducted with the National Centre for Cell Science (NCCS) Institutional Animal Ethics Committee (IAEC) approval of experiments in the NCCS Experimental Animal House (EAH) Facility, and was performed as per the norms, laws and policies laid down by the committee.

### Cell culture, treatments and transfections

Derivation of the A4 progression model of pre-transformed and transformed SeOvCa cells (A4-P and A4-T cells) is described earlier [14], [18]. Retinoid (RXR-γ ligand) treatment was carried out using either natural retinoid *viz*. 9 *Cis* Retinoic acid (CRA;10 µM) or synthetic retinoids Adapalene (ADA; 2 µM; RAR agonist) or 4-[(E)-2-(5,6,7,8 –Tetrahydro - 5,5,8,8 –tetramethyl – 2 naphthalenyl) – 1 -propenyl] benzoic acid Arotinoid acid (TTBPB; 10µM; RXR and RAR agonist) for 48h.

### Sample preparation, 2-Dimensional gel electrophoresis (2DE) and image analyses

Cell pellets (10^7^) of A4-P and A4-T were suspended in 500 µl ml of urea lysis buffer containing 8 M Urea, 2 M Thiourea,100 mM DTT, 2% CHAPS and 0.2% ampholytes with protease-inhibitor cocktail (Amersham USB Guideline). Cell extract was allowed to be mixed for at least 15 minutes and incubated for 30 minutes at room temperature to facilitate proper protein solublisation. Protein samples were further centrifuged (110,000g for 1 hour at 4°C) and suspension was collected. Protein concentration was estimated with 2DE quant kit (GE healthcare) at 480 nm (Bekman Coulter). Prepared samples were run on first dimension (pI) followed by of second dimension in denaturing SDS-PAGE (Mw). A total of 350 µg whole cell protein lysate was taken on 18 cm immobilized pH gradient (IPG) strip (pH 4–7) and rehydrated overnight. A three step IEF voltage program was prepared to the strips on a Protean IEF cell (Bio-Rad): 50 V for 20 mins, 10,000 V for 2 hours minutes and 10,000 V for 45,000 V-hr. Strips were further reduced by incubation in the equilibration/reduction buffer (6 M Urea, 0.375 M Tris pH 8.8, 2% SDS, 20% glycerol, 2% (w/v) DTT (Sigma)) and then alkylated the same buffer but containing 2.5% (w/v) Iodoacetamide (Sigma) instead of DTT. The second dimension was accomplished by running the IPG strips on 1.0 mm-thick of 10%-(w/v)- SDS-PAGE. Gels were stained with mass spectrometry compatible modified coomassie blue (Pierce, Thermo-Fisher) and silver stain. Image acquisition of protein gel was accomplished using Quantity One® software of VersaDocTM system (Bio-Rad Laboratories, USA) with equal parametric values. Image analysis and spot detection were performed on PDQuest 2-DE analysis software advanced version 8.0 (Bio-Rad). For quantitative and qualitative spot comparison across both gels, match-sets (Master set) of six replicates of A4-P and A4-T 2DE-gels were prepared and analysed. Software facilitates annotation of each and individual spot, unique identities were provided to every protein spots of replicate gel. An analysis set of proteins have been prepared to identify spots that are significantly different. Analysis set were made based on identified proteins spots unique to A4-P and A4-T and common spots among two replicate groups with a 2.0 fold quantity variation threshold. A total estimate of matched and unmatched spots was prepared to final images and differential proteins were identified in-between two cell types representative gels.

### In-gel digestion and protein identification using MALDI-TOF/TOF

Protein spots in 2DE showing differential expression and satisfying the statistical criteria were selected and excised for in-gel digestion and further MS analyses. Spot excision was performed manually with the help of sterile sharp spot cutter. Briefly, the gel slices were destained in 25 mM ammonium bicarbonate, subsequent dehydrated with a 2∶1 mixture of 50 mM ammonium bicarbonate:100% acetonitrile (ACN) for repeated 3 times each 5 minutes. Gel slices were reduced with 10 mM DTT at 60°C for 1 hour. After cooling, gel slices were incubated for 15 min at room temperature with 50 mM iodoacetic acid. After washing and dehydrating the gel slices with 25 mM ammonium bicarbonate and ACN for 10 min, they were vacuum dried and tryptic digestion performed with 50 mM ammonium bicarbonate containing 20 ug/mL modified proteomic grade trypsin (Sigma-Aldrich) according to the manufacturer's instructions and kept on ice for 30 min. Additional 25 mM ammonium bicarbonate was added and digestion was continued overnight at 37°C. Extracted peptides were completely dried using a speedvac and re-suspended in 10 µl of 20% Ammonium Bicarbonate and 1% formic acid solution.

After processing through the Zip-Tip pipette tips (Millipore, USA), peptide mixtures were dissolved with matrix solution. The matrix used for MALDI analysis was a-cyano-4-hydroxycinnamic acid (Sigma) at 20 mg/ml in 50% acetonitrile, 0.1% trifluoroacetic acid. Equal volumes of peptide and matrix solution were mixed, and 1 µl of the resulting solution was spotted on a stainless steel MALDI sample plate. Spectra of digested peptides were acquired on a 4800 MALDI-TOF/ TOF mass spectrometer (AB Sciex, Framingham, MA) linked to 4000 series explorer software (version 3.5.3). Produced mass spectra were recorded in a reflector mode within a mass range from 800 to 4000 Da, using a Nd:YAG 355nm laser. The acceleration voltage and extraction voltage were set on 20 kV and 18 kV respectively. Six point calibration of the instrument was performed with peptide standard kit (AB Sciex). All of the MS spectra were obtained from accumulation of 900 shots. MS/MS spectra were acquired with a total accumulation of 1500 laser shots and collision energy of 1Kv. At completion of MS survey scans, the data was processed to generate a list of precursor ions for interrogation by MS/MS. The combined MS and MS/ MS peak lists were explored using the GPS^TM^ Explorer software version 3.6 (AB Sciex). Protein identification was performed by MS/MS ion search using MASCOT (version 2.1) (http://www.martixscience.com) search engine against the SwissProt database. The search parameters were set as follows: all entries and human taxonomy, trypsin digestion and one missed cleavage, fixed modifications: carbamidomethylation of cysteine residues, mass tolerance: 150 ppm for MS and 0.4 Da for MS/MS. Identified proteins having at least two unique matched peptides were selected with an identification confidence interval threshold of ≥95%.

### Co-immunoprecipitation and Immunoblotting

1mg cellular protein extracted in RIPA buffer (1M Tris pH 7.4, 4M NaCl, 0.5M EDTA, NP-40, 10% SDS) was incubated with 5 µg RXR-γ antibody for 2 h at 4°C. This was followed with overnight incubation with 20 µl protein-A agarose (Amersham, GE Healthcare). Complex-bound bead were collected through centrifugation at 12,000 g for 1 min, was washed with TBS (50 mM Tris–HCl, 150 mM NaCl, PMSF), resuspended in 2×SDS buffer and heated at 95°C for 5 min. Eluted proteins were resolved on 2–4% denaturing SDS-PAGE at 80 V followed by immunoblotting, that was performed as described earlier [19]. Details of antibodies used in the study will be made available on request.

### Semi-quantitative reverse transcription-PCR

Semi-quantitative reverse transcription-PCR was performed under standard conditions as described earlier [19] and amplified products resolved on a 1.5% agarose gel; β-actin was used as internal control.

### Cell cycle and apoptosis assay

Cell cycle analysis of transfected and retinoid-treated cells was done with PI (Propidium-Iodide) staining using standard protocols [20]. Data acquisition and analysis was performed on FACSCalibur (Becton Dickinson, San Diego, CA, http:// www.bdbiosciences.com) using ModFit analytical software. Annexin V–FITC apoptosis assay was performed as described earlier [20] using FACSCanto II (Becton Dickinson); DiVa software (Becton Dickinson) was used for data analysis.

### *In vivo* studies

In vivo experimentation was performed in NOD/ SCID mice bred and maintained at Experimental Animal Facility, NCCS; and carried out as per the norms, laws and policies of the institutional ethical committee. A4-T cells (2.5×10^6^) were injected subcutaneously (SC) in thighs of 4–6 week-old male mice and observed every 48 h till 3 weeks for tumor formation. Injections of retinoids i.e. 9Cis RA, ADA and TTNPB as well in combination started while tumor size reaches 25–30 mm^3^ in volume, where DMSO given to vehicle control mice. Treatments of DMSO, 25 µM 9Cis RA, 25 µM 9Cis RA+10 µM TTNPB, 5 µM ADA, 5 µM ADA +10 µM TTNPB and 25 µM 9Cis RA+5 µM ADA +10 µM TTNPB retinoids injections were given twice per week into the tumor of each mouse. Tumor size was monitored in two perpendicular directions using Vernier's calliper; individual tumor weights and sizes were more precisely quantified in the seventh week after sacrificing mice to harvest tumors.

### Statistical analysis

All experiments were carried out at least in triplicate; data are expressed as mean ± SEM of three independent experiments. The significance of difference in the mean values was determined using two–tailed Student's t test; p<0.05 was considered significant. ANOVA test was performed to compare gene and protein expression and tumor volume over time between treatment groups at a significance level of <0.05. Student-Bonferroni test was used to evaluate sub-comparisons to control the test-wise error rate.

## Supporting Information

Figure S1 **A. PI based FACS analysis of cell cycle in A4-P and A4-T cells on different treatment regime of no retinoid treatment, treatment with CRA, ADA and with both having alternative treatment of another synthetic retinoid i.e. TTNPB, showing percentage of relative populations in different cell cycle phases.** B. Quantitation of different cell cycle phases of A4-P and A4-T cells on different retinoid treatments. C. Graphical representation showing tumor volumes of retinoids treated NOD-SCID mice. D. Graphical representation showing tumor weight of retinoids treated NOD-SCID mice. The data shown are representative of three separate experiments and depicted as mean ± SEM *p<0.05, **p<0.01, ***p<0.001.
(DOC)

Table S1 **Summary of total numbers of proteins identified between A4-P and A4-T cells through 2DE analyses followed by MALDI-TOF-TOF (MS/MS) identification.**
(DOC)
