# Supplementary figures and images for: Expression Proteomics Predicts Loss of RXR-γ during Progression of Epithelial Ovarian Cancer
Source: PLoS One. 2013 Aug 6;8(8):e70398. doi: 10.1371/journal.pone.0070398 (PMC3735596; doi:10.1371/journal.pone.0070398)

**Figure S1**

**
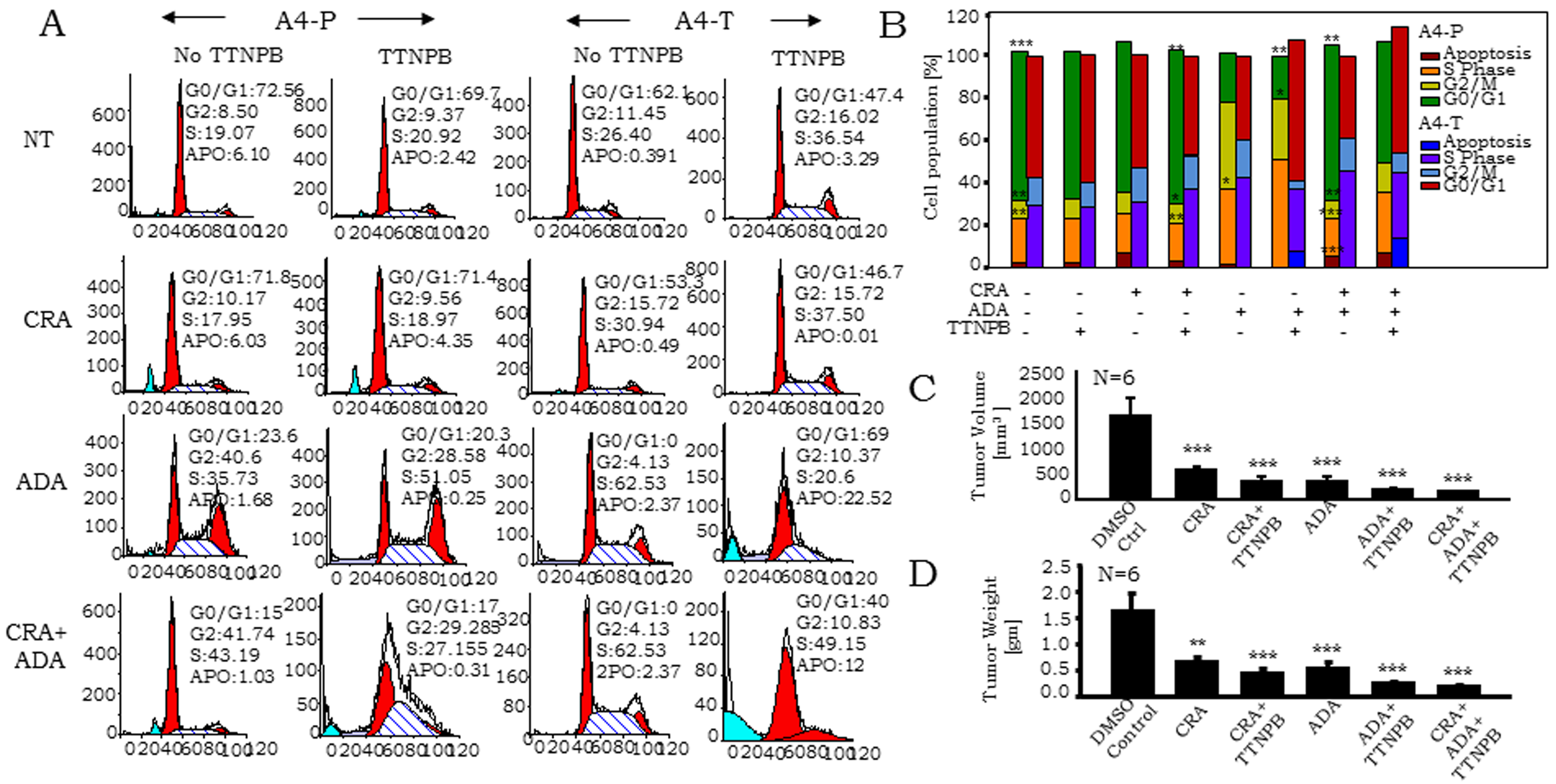
**

Supplement: Figure S1 — A. PI based FACS analysis of cell cycle in A4-P and A4-T cells on different treatment regime of no retinoid treatment, treatment with CRA, ADA and with both having alternative treatment of another synthetic retinoid i.e. TTNPB, showing percentage of relative populations in different cell cycle phases. B. Quantitation of different cell cycle phases of A4-P and A4-T cells on different retinoid treatments. C. Graphical representation showing tumor volumes of retinoids treated NOD-SCID mice. D. Graphical representation showing tumor weight of retinoids treated NOD-SCID mice. The data shown are representative of three separate experiments and depicted as mean ± SEM *p<0.05, **p<0.01, ***p<0.001. (DOC) [file pone.0070398.s001.doc]
