# Supplementary material for: Expression Proteomics Predicts Loss of RXR-γ during Progression of Epithelial Ovarian Cancer
Source: PLoS One. 2013 Aug 6;8(8):e70398. doi: 10.1371/journal.pone.0070398 (PMC3735596; doi:10.1371/journal.pone.0070398)
Supplement: Table S1 — Summary of total numbers of proteins identified between A4-P and A4-T cells through 2DE analyses followed by MALDI-TOF-TOF (MS/MS) identification. (DOC) [file pone.0070398.s002.doc]

| **Table S1 :** Summary of total numbers of proteins identified between A4-P and A4-T cells through 2DE analyses followed by MALDI-TOF-TOF (MS/MS) identification | | | | | | | |
| --- | --- | --- | --- | --- | --- | --- | --- |
| **Group I** | | | | **Group II** | | | |
| **Proteins expressed exclusively in either A4-P or A4-T cells** | | | | **Proteins differentially expressed in both pre-transformed and transformed A4 cells** | | | |
| **Proteins expressed exclusively in A4-P cells** | | **Proteins expressed exclusively in A4-T cells** | | **Upregulated in A4-P cells** | | **Upregulated in A4-T cells** | |
|  | | | | | | | |
| **Protein Name** | **Symbol** | **Protein Name** | **Symbol** | **Protein Name** | **Symbol** | **Protein Name** | **Symbol** |
|  | **10** |  | **34** |  | **31** |  | **48** |
| **Cathapsin D precursor** | **CTSD** | X-ray repair cross-complementing protein 5 | **XRCC5** | Splicing factor, arginine/serine-rich 3 (SRP20) | **SFRS3** | Adenosylhomocysteinase | **AHCY** |
| **Guanine nucleotide-binding protein subunit alpha-11** | **GNA11** | Chloride intracellular channel protein 4 | **CLIC4** | Glutaminase kidney isoform, mitochondrial | **GLS** | Cytokeratin 8 | **KRT8** |
| **Kelch like protein -9** | **KLHL9** | 2-5A-dependent ribonuclease | **RNASEL** | 3-hydroxyisobutyrate dehydrogenase, mitochondrial | **HIBADH** | Gamma actin | **ACTG1** |
| **Myosin IIIa** | **MYO3A** | T-complex protein 1, gamma subunit | **CCT3** | Guanine nucleotide-binding protein G(k) subunit alpha | **GNAI3** | Protein disulfide isomerase A6 | **PDIA6** |
| **Progestrone induced blocking factor-1** | **PIBF1** | eukaryotic translation elongation factor 1 alpha 1 | **EEF1A1** | Guanine nucleotide-binding protein G(I)/G(S)/G(T) subunit beta-1 | **GNB1** | Heat shock protein HSP 90-alpha | **HSP90AA1** |
| **Retinoic acid receptor RXR-gamma** | **RXRG** | Gamma enolase | **ENO2** | Cytochrome b-c1 complex subunit 1, mitochondrial | **UQCRC1** | Spermine synthase | **SMS** |
| **Reticulocalbin 1 precursor** | **RCN1** | Importin subunit alpha-4 | **KPNA4** | NADH ubiquinone oxidoreductase subunit | **NDUFS1** | Importin-5 | **IPO5** |
| **Annexin A5** | **ANXA5** | Nucleophosmin | **NPM1** | Alpha actinin-4 | **ACTN4** | Endoplasmin | **HSP90B1** |
| **Tropomyosin 1 α chain** | **TMP1** | Protein disulfide-isomerase A3 precursor | **PDIA3** | Splicing factor, arginine/serine-rich 1 (SF2/ASF) | **SFRS1** | Major vault protein | **MVP** |
| **Peroxiredoxin 4** | **PRDX4** | Pyruvate kinase isozymes M1/M2 | **PKM2** | Importin subunit beta-1 | **KPNB1** | Heat shock protein HSP 90-beta | **HSP90AB1** |
|  |  | Secernin 1 | **SCRN1** | Serine/threonine-protein kinase tousled-like 2 | **TLK2** | Tubulin-folding cofactor B | **TBCB** |
|  |  | Serum albumin precursor | **ALB** | Tubulin alpha-1B chain | **TUBA1B** | COP9 signalosome complex subunit 4 | **COPS4** |
|  |  | ATP synthase subunit delta, mitochondrial | **ATP5D** | Lamin B1 | **LMNB1** | Eukaryotic translation initiation factor 3 subunit H | **EIF3H** |
|  |  | 26S protease regulatory subunit 6B | **PSMC4** | heterogeneous nuclear ribonucleoprotein K | **HNRNPK** | Elongation factor 1-delta | **EEF1D** |
|  |  | 26S protease regulatory subunit 7 | **PSMC2** | 78 kDa glucose-regulated protein (GRP-78) | **HSPA5** | Poly(rC)-binding protein 2 | **PCBP2** |
|  |  | DNA repair protein RAD50 | **RAD50** | Rho-associated protein kinase 2 | **ROCK2** | Proteasome subunit beta type 4 precursor | **PSMB4** |
|  |  | Elongation factor Tu, mitochondrial | **TUFM** | Ubiquitin-like modifier-activating enzyme 1 | **UBA1** | 14-3-3 protein theta | **YWHAQ** |
|  |  | FK506 binding protein 4, 59kDa | **FKBP4** | 60S acidic ribosomal protein P0 | **RPLP0** | Nuclear transcription factor Y subunit gamma | **NFYC** |
|  |  | 60 kDa heat shock protein, mitochondrial | **HSPD1** | Serine/threonine-protein phosphatase PP1-beta catalytic subunit | **PPP1CB** | eukaryotic translation initiation factor 3, subunit E | **EIF3E** |
|  |  | Peroxiredoxin 2 | **PRDX2** | DJ-1 protein | **PARK7** | Translationally controlled tumor protein | **TPT1** |
|  |  | Ribonuclease inhibitor | **RNH1** | Rab GDP dissociation inhibitor beta | **GDI2** | Cytokeratin 16 | **KRT16** |
|  |  | Heterogeneous nuclear ribonucleoprotein H | **HNRNPH1** | Elongation factor 1-beta | **EEF1B2** | Heat shock protein beta-1 | **HSPB1** |
|  |  | ATP synthase beta chain, mitochondrial precursor | **ATP5B** | Tropomyosin alpha 4 chain | **TPM4** | eukaryotic translation initiation factor 4A1 | **EIF4A1** |
|  |  | Chromobox protein homolog 5 | **CBX5** | F-box/WD repeat-containing protein 1A | **BTRC** | T-complex protein 1 subunit alpha | **TCP1** |
|  |  | Translocon-associated protein subunit delta | **SSR4** | Transportin-1 | **TNPO1** | 14-3-3 protein epsilon | **YWHAE** |
|  |  | Superoxide dismutase [Cu-Zn] | **SOD1** | Alpha-enolase | **ENO1** | Proliferating cell nuclear antigen | **PCNA** |
|  |  | Mago nashi protein homolog | **MAGOH** | Cytokeratin 10 | **KRT10** | Protein SET | **SET** |
|  |  | Myosin Id | **MYO1D** | Lamin B2 | **LMNB2** | Calreticulin precursor | **CALR** |
|  |  | Small nuclear ribonucleoprotein F | **SNRPF** | Diablo homolog, mitochondrial | **DIABLO** | Proteasome subunit beta type-6 | **PSMB6** |
|  |  | Proteasome subunit beta type 3 | **PSMB3** | Vimentin | **VIM** | Cytokeratin 19 | **KRT19** |
|  |  | Beta-2-microglobulin precursor | **B2M** | Sulfotransferase 1A3/1A4 | **SULT1A3** | Erlin-2 | **ERLIN2** |
|  |  | Delta(3,5)-Delta(2,4)-dienoyl-CoA isomerase, | **ECH1** |  |  | Junction plakoglobin | **JUP** |
|  |  | proteasome subunit, alpha type, 1 | **PSMA1** |  |  | Tryptophanyl-tRNA synthetase, cytoplasmic | **WARS** |
|  |  | T-complex protein 1, theta | **CCT8** |  |  | Ezrin | **EZR** |
|  |  |  |  |  |  | RuvB-like 2 | **RUVBL2** |
|  |  |  |  |  |  | Galactokinase | **GALK1** |
|  |  |  |  |  |  | Ornithine aminotransferase, mitochondrial | **OAT** |
|  |  |  |  |  |  | Mitochondrial-processing peptidase subunit alpha | **PMPCA** |
|  |  |  |  |  |  | RuvB-like 1 | **RUVBL1** |
|  |  |  |  |  |  | Annexin A1 | **ANXA1** |
|  |  |  |  |  |  | Protein disulfide-isomerase precursor | **P4HB** |
|  |  |  |  |  |  | Thioredoxin domain-containing protein 4 | **ERP44** |
|  |  |  |  |  |  | Heterogeneous nuclear ribonucleoproteins C1/C2 | **HNRNPC** |
|  |  |  |  |  |  | Calpain-2 catalytic subunit, precursor | **CAPN2** |
|  |  |  |  |  |  | Heat shock 70 kDa protein 1A/1B (HSP70) | **HSPA1A** |
|  |  |  |  |  |  | Cytokeratin 18 | **KRT18** |
|  |  |  |  |  |  | Stress-70 protein, mitochondrial (GRP-75) | **HSPA9** |
|  |  |  |  |  |  | Thioredoxin-dependent peroxide reductase, mitochondrial precursor | **PRDX3** |
